# Rapid identification of porcine bacterial pathogens using an artificial intelligence Raman microbial analyzer for biology

**DOI:** 10.1128/spectrum.01792-26

**Published:** 2026-10-06

**Authors:** Chia-Hung Lu, Ni-Jyun Ke, Chi-Chun Liu, Yu-Hui Liao, Chu Wang, Shih-Wei Liu, Wei-Cheng Chang, Chun-Yi Hsu, Nai-Shun Liao, Yi-Fan Chou, Po-Ren Hsueh, Ming-Tang Chiou, Chao-Nan Lin

**Affiliations:** 1 ITRUST MedTech Inc, Hsinchu, Taiwan; 2 Department of Veterinary Medicine, College of Veterinary Medicine, National Pingtung University of Science and Technology 63279, Pingtung, Taiwan; 3 Animal Disease Diagnostic Center, College of Veterinary Medicine, National Pingtung University of Science and Technology 63279, Pingtung, Taiwan; 4 Sustainable Swine Research Center, National Pingtung University of Science and Technology 63279, Pingtung, Taiwan; 5 Department of Laboratory Medicine, China Medical University Hospital, School of Medicine, China Medical University 38020, Taichung, Taiwan; 6 Division of Infectious Diseases, Department of Internal Medicine, China Medical University Hospital, China Medical University 38020 , Taichung, Taiwan; 7 Department of Laboratory Medicine, National Taiwan University Hospital, National Taiwan University College of Medicine 38020 , Taipei, Taiwan; 8 Department of Internal Medicine, National Taiwan University Hospital, National Taiwan University College of Medicine 38020 , Taipei, Taiwan; Johns Hopkins University, Baltimore, Maryland, USA

**Keywords:** artificial intelligence, swine bacterial pathogens, Raman spectroscopy, veterinary diagnostics, swine pathogen identification

## Abstract

Rapid and accurate identification of bacterial pathogens is critical for effective disease control in pig farms. Conventional bacterial culture-based and molecular diagnostic methods are time-consuming and often impractical for clinical decision-making. Raman spectroscopy, combined with artificial intelligence, offers a label-free alternative and provides isolate-level identification within approximately 2 min. The aim of this study was to evaluate the performance of the commercial Artificial Intelligent Raman Microbial Analyzer for Biology (AIRMAB) for the rapid clinical identification of swine bacterial pathogens. Bacterial isolates were collected from various tissues of diseased pigs between March and November 2025 at the Animal Disease Diagnostic Center, National Pingtung University of Science and Technology, Taiwan. The species-level identification of all the isolates was confirmed using species-specific polymerase chain reaction assays prior to Raman spectral acquisition, ensuring the accuracy of the reference labels. Overall, the AIRMAB system exhibited high performance in the identification of all 14 clinically relevant pig bacterial pathogens. Notably, perfect recall (100%) was achieved for clinically common species in the test set, including *Pasteurella multocida* (*n* = 30) and *Salmonella enterica* serovar Choleraesuis (*n* = 20). In addition, high recall (>90%) was observed for other important bacteria in the test set, including *Escherichia coli* (97%) (*n* = 33), *Streptococcus suis* (94%) (*n* = 35), and *Glaesserella parasuis* (93%) (*n* = 30). This study reports that the AIRMAB system provides rapid, accurate, and label-free identification of swine bacterial pathogens, delivering results within 2 min per isolate, and demonstrating practical value for timely decision-making in routine veterinary diagnostics, especially for common, and clinically important bacterial species.

**IMPORTANCE:**

Bacterial infections pose a major challenge to global swine production, leading to growth retardation, elevated mortality, and substantial production losses. As several swine pathogens are zoonotic, they also pose direct and indirect risks to human health. Traditional diagnostic methods such as polymerase chain reaction are reliable but time-consuming and require well-equipped laboratories. This study introduces the Artificial Intelligent Raman Microbial Analyzer for Biology system, which utilizes Raman spectroscopy and deep learning to identify 14 major swine pathogens in approximately 2 min. This technology offers a highly automated, cost-effective alternative to traditional methods and matrix-assisted laser desorption ionization-time of flight mass spectrometry, facilitating rapid clinical intervention and large-scale screening to safeguard both animal and public health.

## INTRODUCTION

Bacterial infections pose a major challenge to global swine production, particularly in intensive production systems where high stocking densities lead to the rapid transmission of pathogens. The gregarious behavior of pigs further increases the likelihood of disease spread within herds. Common pathogens such as *Streptococcus suis* and *Salmonella* can cause septicemia and various inflammatory diseases in pigs (1), resulting in growth retardation, elevated mortality, and substantial production losses. As pigs are among the most economically important livestock species worldwide, infectious diseases that impair growth performance directly reduce pork productivity and economic output (2). Moreover, several swine pathogens are zoonotic, indicating that pigs may be transmission hosts, thereby posing both direct and indirect risks to human health (3). Consequently, it is essential to identify infectious agents in pigs rapidly and accurately to control disease effectively and safeguard public health.

Conventional diagnostic methods for bacterial infections, including culture-based techniques, bacterial colony morphology, and polymerase chain reaction (PCR) (4), remain the primary diagnostic tools in veterinary medicine. PCR assays provide rapid detection of bacterial nucleic acid. Although these approaches yield reliable results, they are time-consuming, are dependent on the specificity of PCR primer selection, and are expensive. They also require well-equipped laboratories and trained personnel. Raman scattering is caused by incident photons interacting with matter and undergoing energy shifts after electrons are briefly promoted to a virtual state, producing Stokes or anti-Stokes signals that correspond to specific vibrations and rotational transitions. Through these energy-shifted signatures, Raman spectroscopy reveals the characteristic vibrational patterns of molecules, enabling precise structural fingerprinting of diverse materials (5). Owing to its ability to detect subtle biochemical variations with high molecular specificity, Raman spectroscopy has been widely applied across various fields, such as chemical identification and biomedical diagnostics, among others (6, 7).

Raman spectroscopy is a label-free, noninvasive optical technique capable of capturing molecular vibrational profiles from individual cells, thereby enabling direct biochemical and phenotypic assessment without culture-based enrichment (8, 9). This advantage has led to growing interest in the use of Raman spectral features in combination with machine learning methods for identifying microbes. In particular, the integration of Raman data with advanced deep learning architectures, such as convolutional neural networks (CNNs), has been explored to improve classification robustness and species-level discrimination (10–12). Building on these developments, the development of an Artificial Intelligent Raman Detection and Identification System (AIRDIS) was reported in a previous study. The AIRDIS could successfully differentiate gram-negative and gram-positive bacteria in clinical isolates from humans, with accuracies of 98.86% and 97.64%, respectively (13). Furthermore, it exhibited strong performance in identifying common multidrug-resistant bacteria, further supporting the feasibility of Raman-assisted bacterial categorization in practical diagnostic settings (13). However, most previous studies have concentrated on bacterial species identification in samples obtained from humans. The research on the application of this approach to veterinary or animal health is limited.

Building on these developments, the AIRDIS was previously established by our group to differentiate clinical isolates from humans (13). To extend this foundational technology to the veterinary domain, we introduce the Artificial Intelligent Raman Microbial Analyzer for Biology (AIRMAB) in this study. The AIRMAB system utilizes the identical hardware and analytical pipeline as AIRDIS; the distinct nomenclature is primarily employed as a product designation to differentiate its specific application focus on veterinary and general biological fields. Specifically, the aim of the present study is to evaluate the diagnostic performance of the AIRMAB system for the rapid identification of clinically important bacterial pathogens in pigs.

## MATERIALS AND METHODS

### Study design

**Fig 1:**
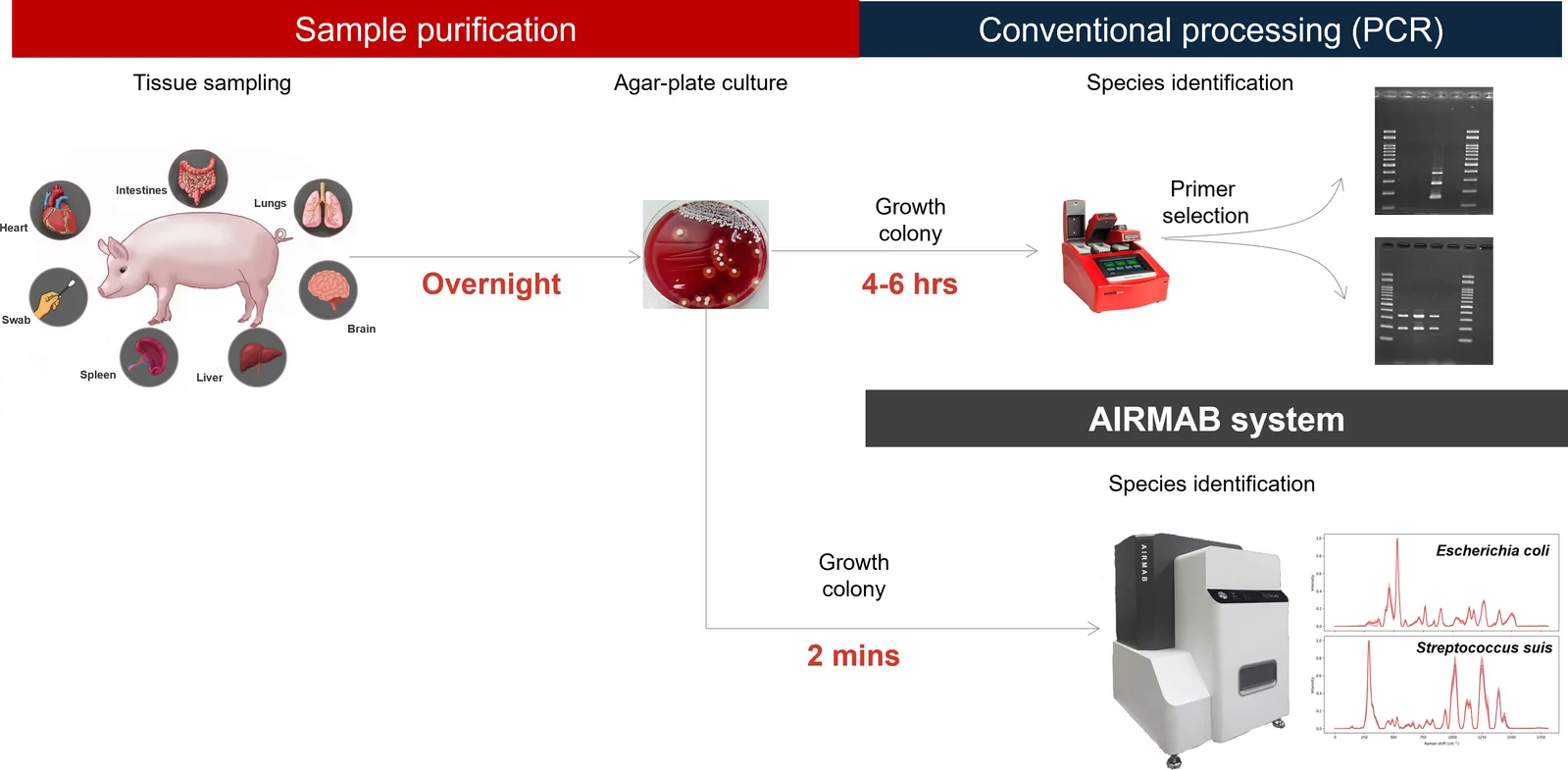
Workflow of swine bacterial culture and identification in clinical isolates from diseased pigs using conventional processing and the AIRMAB system, respectively.

The AIRMAB system was developed, and a single-center, laboratory-based validation study was conducted at the Animal Disease Diagnostic Center, National Pingtung University of Science and Technology, between March and November 2025 to evaluate its performance. Clinical specimens were collected from diseased pigs and cultured on agar plates overnight to obtain purified isolates. Following incubation, a well-isolated colony from each plate was selected for downstream analyses as shown in Fig. 1. Each purified isolate was processed in parallel for conventional PCR-based identification, while bacterial spectral data were collected using the AIRMAB system developed in this study for species identification.

### Bacterial isolation and molecular identification of bacterial species

Clinical specimens were obtained by licensed veterinarians as part of routine diagnostic procedures. Before sampling, the tissue surface was sterilized with a heated cautery device, and the target tissue was aseptically excised for analysis. Molecular identification of bacterial species was conducted using a PCR assay. Presumptive identification was first performed by the attending veterinarian, after which PCR assays using species-specific primers were conducted by trained laboratory personnel to confirm the identification of bacterial species. The PCR results served as the reference standard for evaluating the identification and diagnostic performance of the AIRMAB system. Table 1 summarizes the PCR assay for different bacterial isolates in this study.

**TABLE 1:** Summary of PCR assay for different bacterial isolates in this study

Pathogen	Research title	References
*Escherichia coli*, Hemolytic *E. coli*	Molecular characterization and antibiotic resistance of enterotoxigenic and entero-aggregative *Escherichia coli* isolated from raw milk and unpasteurized cheeses	(14)
	Comparison of virulence gene profiles of *Escherichia coli* strains isolated from healthy and diarrheic swine	(15)
*Salmonella* species	Rapid identification of *Salmonella* enterica serovars, Typhimurium, Choleraesuis, Infantis, Hadar, Enteritidis, Dublin and Gallinarum, by multiplex PCR	(16)
*Pasteurella multocida*	Development of PCR assays for species- and type-specific identification of *Pasteurella multocida* isolates	(17)
*Haemophilus parasuis* (*Glaesserella parasuis*)	Development of a multiplex PCR assay for rapid molecular serotyping of *haemophilus parasuis*	(18)
*Actinobacillus pleuropneumoniae*	Detection of *Actinobacillus pleuropneumoniae* in cultures from nasal and tonsillar swabs of pigs by a PCR assay based on the nucleotide sequence of a dsbE-like gene	(19)
*Streptococcus suis*	Development of an appropriate PCR system for the reclassification of *Streptococcus suis*	(20)
*Streptococcus* species	Use of tuf sequences for genus-specific PCR detection and phylogenetic analysis of 28 *Streptococcal* Species	(21)
*Staphylococcus hyicus*	Species identification and strain typing of *Staphylococcus agnetis* and *Staphylococcus hyicus* isolates from bovine milk by sse of a novel multiplex PCR assay and pulsed-field gel electrophoresis	(22)
*Clostridium perfringens* Type A and Type C	Diagnostic multiplex PCR for toxin genotyping of *Clostridium perfringens* isolates	(23)
*Clostridium difficile*	Quantification of *Bifidobacterium* spp., *Escherichia coli* and *Clostridium difficile* in fecal samples of breast-fed and formula-fed infants by real-time PCR	(24)
*Erysipelothrix rhusiopathiae*	Characterization of *Erysipelothrix rhusiopathiae* strains isolated from recent swine erysipelas outbreaks in Japan	(25)
*Trueperella pyogenes*	Antimicrobial susceptibility of *Trueperella pyogenes* isolated from food-producing ruminants	(26)

### Single-isolate Raman measurements using the AIRMAB platform

For sample pretreatment, intact bacterial colonies were mixed with silver nanoparticle substrates for surface-enhanced Raman spectroscopy (SERS) and deposited onto a stainless-steel plate (ITRUST MedTech Inc.) to air-dry (13). This preparation process enables the acquisition of an enhanced Raman signal for downstream identification (27). After mixing with the SERS substrate, the samples were dried into circular spots and subsequently loaded into the AIRMAB system for spectral acquisition. Raman measurements were performed using a 785-nm excitation laser with a laser power of 500 mW at the sample surface and an integration time of 1 s per spectrum to induce scattering from the bacterial cells. For each sample spot, spectra were collected from ten randomly selected locations, with duplicate readings taken at each location to ensure measurement consistency.

All Raman spectra were subjected to a standardized preprocessing workflow prior to analysis. Baseline correction was performed using a polynomial fitting algorithm (order = 5) to eliminate background interference, followed by Savitzky–Golay smoothing (window size = 31 and polynomial order = 5) to attenuate high-frequency noise while preserving intrinsic spectral features. This preprocessing strategy ensured the maintenance of the linear dependencies between spectral intensity, laser power, and integration time, thereby enhancing overall signal stability. Subsequently, intensity normalization was applied to rescale all the spectra to a common numerical range, ensuring comparability across samples and facilitating downstream modeling (13, 28).

### Model architecture and training

We employed the Raman Net architecture for bacterial strain classification (29), while the overarching model design followed the framework established in our previously published work (13). In this approach, each Raman spectrum was segmented into multiple overlapping fragments, and each fragment was independently normalized and processed through a multilayer perceptron (MLP) to extract localized spectral features. The features from all the fragments were concatenated to create a global representation of the spectrum, followed by regularization using a dropout layer. We employed a composite model architecture that integrates a convolutional neural network (CNN)-based Raman Net for spectral feature extraction with a multilayer perceptron (MLP) for final bacterial strain classification. In this framework, the parameters of the Raman Net feature extractor were fixed, and the extracted features were concatenated and processed through the MLP. The model was trained using standard empirical hyperparameter settings, specifically, a batch size of 64, 80 epochs, and a learning rate of 0.00001, without performing a grid search for further parameter tuning. The final optimized Keras model comprises 473,738 total parameters, with 470,270 trainable parameters in the MLP layers. To evaluate the model’s generalizability and prevent data leakage, the data set was randomly partitioned strictly at the isolate level into training and test sets at a ratio of 7:3. All training and evaluation were performed using TensorFlow (version 2.9.1), Keras (version 2.9.0), and scikit-learn (version 1.3.2).

### Model performance evaluation

Since 20 independent Raman spectra were collected per isolate, the final isolate-level prediction was determined by calculating the mean prediction probability. Specifically, the model output probabilities for each class were averaged across all 20 spectra, and the bacterial species with the highest average probability score was assigned as the final predicted label for that isolate. Furthermore, to address the limitation of closed-set classification and prevent confident misclassifications of out-of-distribution (OOD) samples, such as untrained species, a confidence thresholding mechanism was implemented. If the highest mean prediction probability for an isolate was below 0.60, the system refrains from assigning a species label. Instead, it outputs a “Not reliable” warning, advising the user that the prediction is for reference only and recommending a re-test or verification of sample quality. Model performance was then assessed using three standard classification metrics: precision, recall, and F1-score. These metrics were computed for each bacterial class and summarized using macro-averaging to ensure equal weighting across classes.

## RESULTS

### Collection of clinical isolates

**Fig 2:**
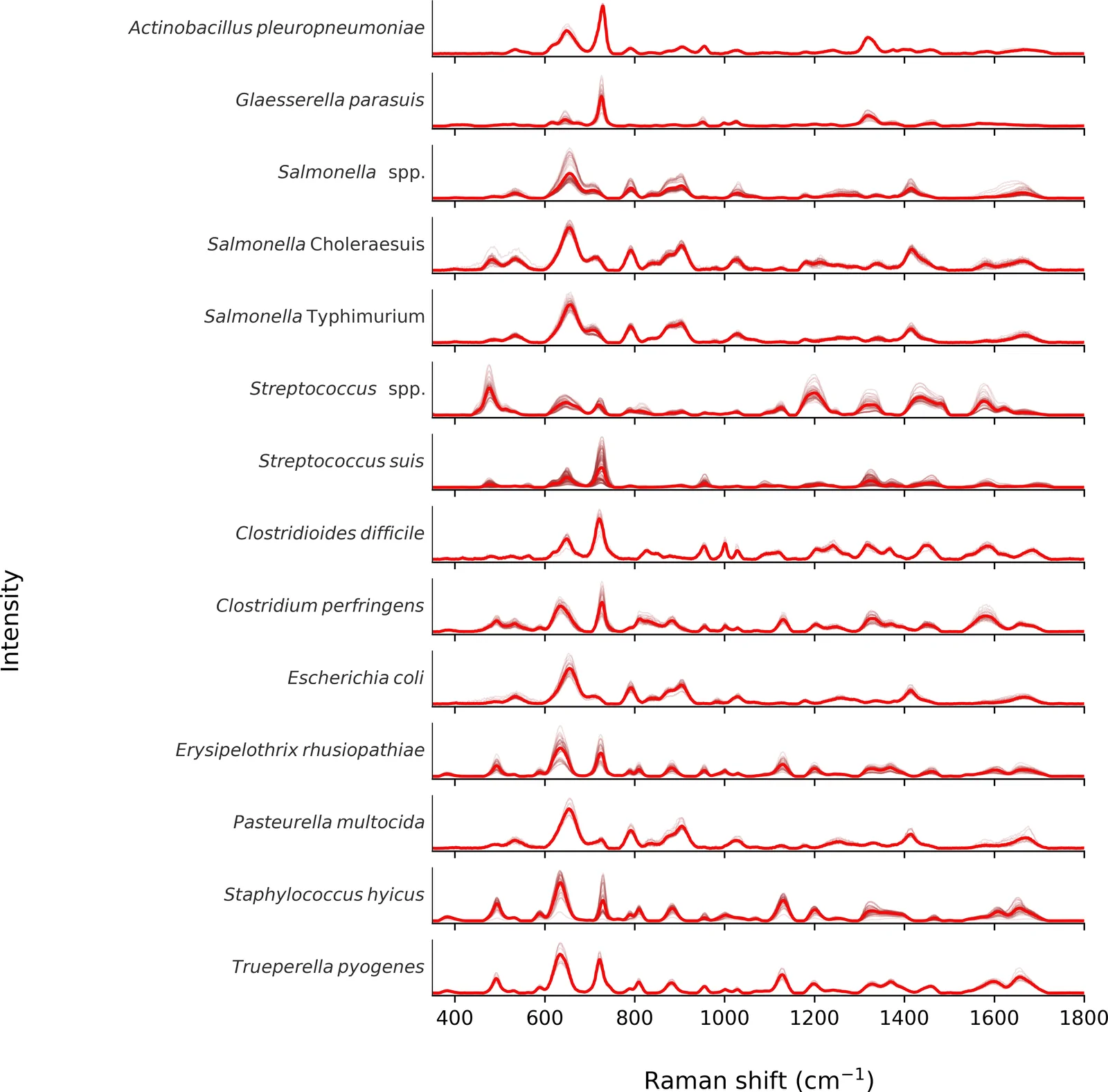
Per-species average Raman spectra of the 14 swine bacterial pathogens.

A total of 840 clinical bacterial isolates representing 14 frequently detected swine pathogens were included, namely *Actinobacillus pleuropneumoniae* (*n* = 32), *Clostridioides difficile* (*n* = 58), *Clostridium perfringens* (*n* = 40), *Escherichia coli* (*n* = 110), *Erysipelothrix rhusiopathiae* (*n* = 11), *Glaesserella parasuis* (*n* = 101), *Pasteurella multocida* (*n* = 100), *Salmonella enterica* serovar Choleraesuis (*S*. Choleraesuis) (*n* = 67), *Salmonella enterica* serovar Typhimurium (*n* = 52), *Salmonella* spp. (*n* = 56), *Streptococcus suis* (*n* = 118), *Streptococcus* spp. (*n* = 64), *Staphylococcus hyicus* (*n* = 16), and *Trueperella pyogenes* (*n* = 15). The data set was randomly divided at a 7:3 ratio, yielding 588 isolates for training and 252 isolates for testing. The species distribution within the test set is presented in Table 2. All the isolates were confirmed by veterinary evaluation and PCR assays, which served as the gold standard. Raman spectra were subsequently acquired for each isolate using the AIRMAB platform, with a minimum of twenty spectra collected per isolate. The spectra of the 14 bacterial species are shown in Fig. 2.

**TABLE 2:** Classification performance of the AIRMAB system

Bacteria	No. of isolates (%)	Metrics (%)		
		Precision	Recall	F1-score
*Trueperella pyogenes*	5 (1.98)	100.00	100.00	100.00
*Glaesserella parasuis*	30 (11.9)	96.55	93.33	94.92
*Salmonella enterica* serovar Choleraesuis	20 (7.94)	100.00	100.00	100.00
*Streptococcus* spp.	20 (7.94)	90.48	95.00	92.68
*Escherichia coli*	33 (13.1)	96.97	96.97	96.97
*Staphylococcus hyicus*	5 (1.98)	100.00	100.00	100.00
*Erysipelothrix rhusiopathiae*	3 (1.19)	100.00	100.00	100.00
*Clostridioides difficile*	17 (6.75)	100.00	100.00	100.00
*Streptococcus suis*	35 (13.89)	100.00	94.29	97.06
*Salmonella enterica* serovar Typhimurium	15 (5.95)	93.75	100.00	96.77
*Salmonella* spp.	17 (6.75)	100.00	100.00	100.00
*Pasteurella multocida*	30 (11.9)	100.00	100.00	100.00
*Actinobacillus pleuropneumoniae*	10 (3.97)	90.91	100.00	95.24
*Clostridium perfringens*	12 (4.76)	100.00	100.00	100.00

### Performance of the AIRMAB in bacterial identification

The AIRMAB system, which integrates Raman spectroscopy with a multiclass deep learning model, exhibited a strong identification capability across all 14 bacterial species. The precision, recall, and F1-scores for each bacterial species, categorized by class, are summarized in Table 2. Species represented by more than 10 isolates, such as *C. difficile*, *C. perfringens*, *P. multocida*, *S*. Choleraesuis, and *Salmonella* spp. achieved perfect classification, with the precision, recall, and F1-score all reaching 100%. These findings highlight the system’s reliability and robust predictive capability for clinical isolates.

**Fig 3:**
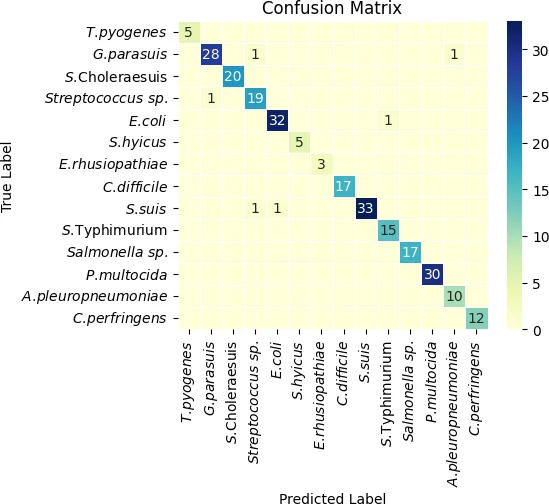
Confusion matrix for the 14-class bacterial species identification in the test set.

In addition, for species with larger sample sizes (*n* > 30), such as *E. coli*, the model performed consistently well, with all three metrics exceeding 96%. Although this performance was not perfect, the balanced distribution of precision, recall, and F1-score reveals that the model effectively discriminates this species while allowing only minimal misclassification. The confusion matrix (Fig. 3) supports this observation, revealing that for *E. coli*, only a single isolate was misclassified into another category.

With respect to *S. suis*, which had a relatively large sample size, the model reached a level of 100% precision, with a recall and F1-score of 94.29% and 97.06%, respectively. The perfect precision indicates that all the predicted *S. suis* isolates were correctly classified. However, the slightly lower recall reflects that a few true *S. suis* isolates were misassigned to other classes. Overall, the AIRMAB system exhibited stable performance for species with large sample sizes.

## DISCUSSION

In our study, we demonstrated that the AIRMAB system exhibited high identification performance for all 14 clinically relevant bacterial pathogens in swine. The AIRMAB consistently achieved high precision, recall, and F1-scores for species with relatively large numbers of isolates, demonstrating reliable and stable classification performance under well-represented conditions. Notably, species such as *P. multocida* and *S*. Choleraesuis achieved perfect recall. The latter is of particular importance, as *S*. Choleraesuis is a clinically significant swine-associated serovar that is frequently associated with systemic infection, particularly septicemia, resulting in substantial growth retardation and mortality in pig populations (30, 31). Historical surveillance in Taiwan has revealed that it was once the most isolated *Salmonella* serotype in clinically affected pigs, reflecting its high prevalence (32, 33). In addition to its veterinary relevance, *S*. Choleraesuis is recognized as a highly invasive zoonotic pathogen that can cause species and extraintestinal disease in humans (34). Additionally, high recall values (>90%) were achieved for other clinically relevant species, including *E. coli* (96.97%), *S. suis* (94.29%), and *G. parasuis* (93.33%). These pathogens are common infectious agents in swine and are associated with multiple diseases, such as meningitis, endocarditis, septicemia, and bronchopneumonia, and contribute substantially to economic losses in the swine industry (35, 36). While the model achieved perfect 100% precision and recall for several species, we acknowledge that for classes with a very limited number of isolates in the test set (e.g., *Erysipelothrix rhusiopathiae*, *n* = 3; *Staphylococcus hyicus*, *n* = 5), this perfect classification may partially reflect the limited intra-species variance currently captured in our data set rather than true perfect generalization. Future studies incorporating a larger, more diverse collection of these specific clinical isolates will be necessary to fully validate the perfect classification performance.

Given the substantial clinical consequences and economic losses associated with these pathogens, the development of diagnostic tools capable of rapid and correct identification is critically important. In this context, the AIRMAB system offers a practical solution by integrating Raman spectroscopy with deep learning to enable high detection performance of microbial strains. Raman spectroscopy captures molecular-level vibrational signatures that serve as unique biochemical fingerprints for different bacterial species, whereas the deep learning framework effectively extracts and interprets these high-dimensional spectral features. By amplifying subtle inter-strain differences, the model enhances classification performance and supports reliable species-level discrimination. Moreover, Raman-based analytical approaches have also been successfully used in various clinical diagnostics settings (37).

In routine diagnostic settings for pigs, PCR-based pathogen identification typically requires several steps, such as DNA/RNA extraction, amplification, and subsequent analysis. Matrix-assisted laser desorption ionization-time of flight mass spectrometry (MALDI-TOF MS) is also widely employed in clinical microbiology laboratories, though it typically involves distinct instrument and maintenance considerations. As demonstrated in this study, the AIRMAB system provides a highly automated, rapid analytical pipeline that serves as a label-free alternative. Based on our estimates, the initial setup cost (<USD 134,000) and annual maintenance expenses (USD 10,000) for the AIRMAB system are highly competitive compared to standard MALDI-TOF MS systems (USD 300,000), alongside a more economical per-sample testing cost (AIRMAB: USD 2.5 vs MALDI-TOF MS: USD 8). Furthermore, while the overall clinical workflow still requires an overnight culture to obtain pure isolates, the AIRMAB system can generate bacterial identification results with an analytical turnaround time of approximately 2 min once the isolate is loaded (Fig. 1). Rather than making direct performance comparisons with conventional molecular detection methods, which depend on specific primer selection (38, 39), we emphasize that this Raman-based approach offers a complementary tool that reduces operational complexity and enhances the feasibility of timely clinical application and large-scale screening in swine production systems.

Through this evaluation, the feasibility and applicability of Raman-assisted bacterial detection in veterinary and livestock health settings were explored in this study. Our study incorporated a broad range of clinically common swine bacterial pathogens, ensuring that the evaluation reflected real-world conditions. However, a few species were represented by relatively limited numbers of isolates. Continued collection of these less common species will be essential to further strengthen the model’s performance and ensure reliable identification across all pathogen classes. Additionally, a common challenge in clinical applications is the closed-set nature of deep learning models, which may confidently misclassify untrained species or mixed cultures. To address this, while the core neural network is trained on a closed set of 14 species, the integration of the 0.60 probability threshold serves as a practical rejection option for out-of-distribution (OOD) samples or low-quality spectra. This built-in fail-safe significantly mitigates the risk of false-positive identification in real-world field specimens, further supporting the reliability of the AIRMAB system for routine diagnostic and large-scale screening applications.

In summary, this study demonstrates the successful extension of our previously established Raman-based platform to the veterinary domain. By applying this technology to swine bacterial pathogens via the AIRMAB system, we highlight its practical value for the accurate identification of veterinary clinical isolates. Although the overall clinical turnaround time still encompasses an overnight agar culture step to isolate pure colonies from field specimens, the AIRMAB platform delivers results within 2 min per isolate. This is particularly useful for timely decision-making in routine veterinary diagnostics, especially for common and clinically relevant species.

## Data Availability

The original contributions presented in the study are included in the article; further inquiries can be directed to the corresponding authors.
